# Medical Opinion and Movement

**Published:** 1907-09-14

**Authors:** 


### Medical Opinion and Movement.

The view that caisson disease is due to the liberation of bubbles of gas from the tissues in passing from high to low atmospheric pressures, receives confirmation from the experimental work of Dr. Horace M. Vernon. He has found that the solubility of oxygen and nitrogen in fats of such different constitution as olive oil, cod-liver oil, and lard is practically the same, and that it does not vary with the temperature within the range of 15° to 37° C. He is justified, therefore, in assuming that human fat would yield the same results. Taking the mean of the figures obtained for the solubility of oxygen and nitrogen in these fats, he finds that oxygen is 4.5 times more soluble and nitrogen 5.3 times more soluble than in water. As blood plasma dissolves less nitrogen than an equal quantity of water, human fat may be assumed to dissolve at least five times as much nitrogen as the blood. The commonest symptoms of caisson disease are joint and muscular pains and paralysis, and necropsies on caissoniers have shown softening of the spinal cord in the dorsal region, signs of myelitis, and small fissures in the mid-dorsal region. It seems probable, therefore, that the pathology of the disease is explained by the sudden liberation of gas absorbed by tissues like the spinal cord, peripheral nerves, and yellow-bone marrow, which contain a large proportion of fat in their constitution.

The effect of gases in the tissues has been studied by M. Pierre Delbet from another point of view. It is generally supposed that the introduction of air into the veins in the course of any surgical procedure is highly dangerous, and M. Delbet has carried out some experiments on dogs to ascertain how far this is true. He finds that air injected intravenously is easily absorbed by the blood, and so long as it is able to do this there are no untoward effects. The air is carried by the blood to the right side of the heart and thence to the lungs, which act as the organs of elimination. Any injurious effects are purely mechanical, and depend upon the rate at which the air enters the blood. Any quantity of air can be injected provided the rate is not allowed to exceed a certain limit. He finds by experiment that for dogs this rate should be about 5 c.c. of air per minute per 5 kilogrammes body weight. According to this author it is not the cardiac distension which determines death. Respiration is arrested before the heart-beat. M. Delbet suggests that the introduction of oxygen intravenously might be of service in cases of chloroform poisoning, and possibly also in cases of general infections.

It seems unfortunately possible that the New Sydenham Society, which will celebrate its jubilee next year, may at the same time become defunct. The chief reason for such a *dénouement* is lack of funds for carrying on the work. At the annual meeting, which was held during the progress of the British Medical Association at Exeter, the Council submitted a report in which they recommended that the affairs of the Society should be wound up. Mr. Jonathan Hutchinson, however, who has for some time been closely associated with the Society, made certain alternative proposals, to the effect that the Society should work on more restricted lines. A volume of monographs, papers or lectures translated from foreign sources should be published every year. The publication of the *Atlas* should be continued, but the plates reduced in size and the letterpress curtailed. He proposed further that arrangements might be made with the British Medical Association and also with the Royal Society of Medicine for the reproduction of plates in the *Atlas*, which would be to the advantage of the Society. An endeavour should also be made to render it more international in character and bring it into closer relation with all English-speaking communities. The alternative propositions will be put to the members, and the decision of the question will rest with them. Although there has been a shrinkage in membership, the Society still boasts nearly 900 members, and the accounts show that it is up to the present solvent. If, therefore, the interest of members in the Society is still alive we may hope to see it take a fresh lease in life. It has done such good work in the past that, apart from sentiment, it would be a matter of great regret if that work should now suddenly cease.

The pathologist, be he bacteriologist, hæmologist, or microscopist, is no longer shut up in the post-mortem room or laboratory. His services are in constant request for the physician at the bedside and the surgeon in the operating theatre. Time was when his province lay for the greater part in research on the cadaver, and his work only served to guide the future action of the clinician. Now he is a necessity at every step, and he constantly determines the immediate diagnosis and treatment of the patient. This growing importance of the pathologist is well emphasised in an excellent address by Mr. C. B. Lockwood, of St. Bartholomew's Hospital. He points out that we are no longer justified in accepting such expressions as cellulitis, vulvitis, cystitis, and the like as sufficient diagnosis of morbid conditions. We must search further, take cultures, and discover the micro-organism giving rise to such conditions in individual cases. Apart from the employment of sera and vaccines, the determination of the causal microbe may seriously modify both the prognosis and treatment. In the case of cancer, too, a certain diagnosis can be made in many cases only through the microscope, and he condemns the indiscriminate removal of tumours, particularly of breast tumours, on a diagnosis based upon physical signs. He pleads for the presence of the pathologist with the freezing microtome in the operating theatre for all such cases, so that the diagnosis can be definitely determined on the spot and the operation modified in accordance with the finding. He cites a case in which a tumour, supposed to be only a chronic mammary tumour, was removed from one breast and was found afterwards to contain cancer cells. Six months later a similar tumour appeared in the other breast. It seemed natural to suppose that this was certainly cancer, and a sweeping operation was performed. This time, however, it turned out to be only a simple chronic mammary tumour!

